# 

**Published:** 1887-12

**Authors:** 


1845. ★ 1887.

### Ohio College of Dental Surgery.

### CINCINNATI, OHIO.

### SESSION 1887-8.

### FACULTY.

J. S. CASSIDY, M.D., D.D.S., *Professor of Chemistry and Materia Medica.*
H. A. SMITH, D.D.S., DEAN OF THE FACULTY, *Professor of Operative Dentistry and Dental Pathology.*
C. M. WRIGHT, D.D.S., *Professor of Physiology and General Pathology.*
WM. KNIGHT, M.D., *Professor of Anatomy and Oral Surgery.*
GRANT MOLLYNEAUX, D.D.S., *Professor of Mechanical Dentistry and Metallurgy.*

GEO. W. KEELY, D.D.S., *Lecturer on Irregularities of the Teeth.*
OTTO ARNOLD, D.D.S., *Lecturer on Anæsthetics.*

### DEMONSTRATORS.

A. T. OLMSTED, D.D.S., *Demonstrator of Operative Dentistry.*
B. C. HINKLEY, D.D.S., *Ass't Demonstrator of Operative Dentistry.*
JAS. SILCOTT, D.D.S., *Demonstrator of Mechanical Dentistry.*
G. S. JUNKERMAN, M.D., D.D.S., *Demonstrator of Analytical Chemistry.*
H. C. MATLACK, D.D.S., *Demonstrator of Anatomy.*
C. H. MARTIN, D.D.S., *Assistant to Department of Orthodontia.*

The Forty-Second Annual Winter Session begins Tuesday, October 4, 1887, and continues uninterruptedly through five months.

At the close of the regular Session a Spring Course of practical instruction will be inaugurated.

Requirements for Admission and Graduation are those adopted by the National Association of Dental Faculties.

### TUITION FEES:

| | |
|---|---|
| Matriculation Fee (but once) | $ 5 00 |
| Professors' Tickets for first session | 75 00 |
| Professors' Tickets for second year | 50 00 |
| Dissecting Ticket, including material | 10 00 |
| Analytical Chemistry | 10 00 |
| Diploma Fee | 25 00 |

**For further information and announcement, address,**

**H. A. SMITH, DEAN,**

**128 Garfield Place, Cincinnati, O.**

### Northwestern College of Dental Surgery

**SOUTHEAST COR. WABASH AV. AND TWELFTH STREET,**

CHICAGO, ILL.

### BOARD OF DIRECTORS.

H. C. MAGNUSSON, President.
N. J. ROBERTS,
F. H. B. McDOWELL, Secretary.

### BOARD OF EDUCATIONAL CONTROL.

J. E. HEQUEMBOURG, M.D., President. A. P. MERRILL, D.D.S.
JOSEPH HAVEN, M.D. N. J. ROBERTS, D.D.S.
BYRON D. PALMER, D.D.S.

### SUPERVISORS OF THE CLINIC.

M. Stout, D.D.S. A. P. Merrill, D.D.S. Byron D. Palmer, D.D.S.

### FACULTY.

G. C. PAOLI, A.M., M.D., Emeritus Professor of Materia Medica.
N. P. PEARSON, A.M., M.D., Emeritus Professor of Pathology.

JOSEPH HAVEN, M.D., Professor of Physiology and Dean of the Faculty.
A. P. MERRILL, D.D.S., Professor of Operative Dentistry & Dental Histology.
BYRON D. PALMER, D.D.S. Professor of Prosthetic Dentistry.
M. STOUT, D.D.S., Professor of Clinlcal Dentistry.
NORMAN J. ROBERTS, D.D.S., Professor of Oral Surgery.
J. E. HEQUEMBOURG, M.D., Professor of Anatomy and Principles and Practice of Surgery and Surgical Pathology.
F. C. CALDWELL, M.D., Professor of Materia Medica.
J. H. SALISBURY, M.D., Professor of Chemistry.
J. H. LYON, M.D., Professor of Dental Pathology.

### DEMONSTRATORS.

T. C. RIVERA, M.D., - - - Demonstrator of Chemistry.
Dr. G. F. SCHAFER, Jr., - Demonstrator of Mechanical Dentistry.
Dr. L. E. IRELAND, - - Demonstrator of Operative Dentistry.

### Announcement for the Course of 1887-8.

On Tuesday, October 4, 1887, the Northwestern College of Dental Surgery commences its Third Annual Session; and, in the interest of advanced education in the Dental Profession, the Directors have decided to extend the school year from the present course of six months to one of nine months, thus giving a longer time for study and practical instruction and avoiding the crowding necessary in a shorter term.

From its first foundation this College has succeeded far beyond the hopes of its founders and the most sanguine expectations of its friends, and the number and character of its students have exceeded all anticipations. While we feel justified in saying that from its organization the facilities and opportunities for securing a dental education in it have been equal to any other similar institution of learning in the country, we are equally aware that the profession and the people would justly have more confidence in the scholarship and capabilities of graduates of a dental college that requires two terms of nine months' each, than they would have in the graduates of a college that requires two terms of but four or five months' attendance at most, with a preliminary term of a month or two, the attendance upon which is optional with the student. The motive for this change is one only of professional and public interest; but we expect it will meet with the commendation of both. The outlines of the course of instruction, which is a graded one so arranged as to take the student step by step through all the branches of medical and dental theory and practical work to a high standard of profession skill, will be found in the annual announcement, which will be furnished upon application.

The clinics of this College—owing to its favorable location, and the high reputation attained by its clinical professors and demonstrators in the treatment of oral diseases, and the minor operations in dentistry—are the most largely attended of any of the dental colleges of the country; and the infinite variety of operations which a population of over 700,000 offers, renders the experience gained in the clinic rooms of this College invaluable to the student and practitioner.

Every candidate for admission must be eighteen years of age, and must present to the Faculty satisfactory evidence of a good moral character. Unless already a matriculate of the College, or of some other recognized college or university, or a graduate of some recognized academy or high school, or holding a teacher's certificate, he must pass an examination in Arithmetic, Geography, English Grammer, and Composition, United States History, and Natural Philosophy. (After July 1, 1887, in Elementary Chemistry.) He shall subscribe to Article II., Section 3, of the Code of Ethics of the American Dental Association.

The candidate for the degree of Doctor of Dental Surgery must have attained the age of twenty one years. He shall have passed a satisfactory examination, both oral and written—a written examination being substituted in this College for a thesis. He shall have studied dentistry three years, including two courses of lectures, one of which shall be at this institution. Graduates in medicine may apply for the degree of D.D.S., after having had two full years of practical instruction or experience in dentistry, one year of which, including one course of lectures, must be spent in the College. After these requirements have been complied with, upon recommendation of the Faculty and approval by the Board of Directors, the candidate will receive the degree of Doctor of Dental Surgery.

### FEES.

For the first year a student is a member of the college the fee is $100, payable in two installments of $75 and $25; for the second year $100, in payments of $60 and $40; for any subsequent year, $50. There are no other fees, either for matriculation, demonstration, or diploma, the above covering all the tuition and graduation fees of the complete course. For information, address

F. H. B. McDOWELL, *Secretary*,
1201 Wabash Avenue, Chicago, Ill.

### LOUISVILLE

### COLLEGE OF DENTISTRY,

*Dental Department of Central University,*

**LOUISVILLE, KY.**

THE REGULAR SESSION OF THIS INSTITUTION BEGINS JANUARY 20, 1887, AND ENDS JUNE 17, 1887.

### FACULTY.

A. WILKES SMITH, M.D., D.D.S., Professor of Oral and Dental Surgery and Operative Dentistry. Dean of Faculty.

CHARLES G. EDWARDS, D. D. S., Professor of Prosthetic and Clinical Dentistry.

A. M. CARTLEDGE, M.D., Professor of Surgery.

DUDLEY S. REYNOLDS, M.D., Professor of Pathology and Hygiene.

FRANK C. WILSON, M. D., Professor of Principles and Practice of Medicine.

SAMUEL G. DABNEY, M. D., Professor or Physiology and Histology.

JOHN A. LARRABEE, M. D., Professor of Materia Medica and Therapeutics.

C. SKINNER, M. D., Professor of Anatomy.

JAS. LEWIS HOWE, M. D., Ph. D., F. C. S., Professor of Medical Chemistry and Toxicology. Scientist to the Polytechnic Society of Kentucky.

### DEMONSTRATORS.

H. B. TILESTON, D. D. S., Demonstrator of Operative Dentistry.

JOHN H. BALDWIN, D. D. S., Demonstrator of Prosthetic Dentistry.

GEORGE F. MARTIN, M. D., Demonstrator of Anatomy.

### FEES.

| | |
|---|---|
| Matriculation, | $5 00 |
| Demonstrations in Anatomy, | 10 00 |
| Lectures, | 75 00 |
| Graduation, | 30 00 |
| Hospital Ticket (required by city), | 5 00 |

### DIRECTORY OF DENTAL SOCIETIES.

*American Dental Association*—Meets on the 1st Tuesday of August, 1888, at Louisville, Ky. Pres. Frank Abbott, New York; Cor. Sec. Lewis Ottofy, Chicago; Rec. Sec. Geo. H. Cushing, Chicago.

### STATE DENTAL SOCIETIES.

*Alabama Dental Association*—Meets annually. Next meeting at Selma, on the second Tuesday of April, 1888. Pres. T. P. Whitby, Wetumpka; Sec. T. M. Allen, Birmingham.

*California State Dental Association*—Third Tuesday in July, 1888. Pres. W. F. Griswold, Grass Valley; Rec. Sec. W. A. Knowles, San Francisco; Cor. Sec. W. Z. King, San Francisco. Next Meeting in San Francisco.

*Connecticut State Dental Society*—Pres. E. A. Stebbins, Shelburne Falls; Rec. Sec. Geo. A. Maxfield, Holyoke. Annual meeting Oct. 27th and 28th, 1887, at Hartford.

*Dental Society of the State of Maryland and District of Columbia*—Meets annually. Next meeting at Washington City, on the second Tuesday in October, 1888. Pres. B. F. Coy, of Baltimore: Sec. M. W. Foster, M. D.

*Georgia State Dental Society*—Meets first Tuesday in August, 1888, at Cumberland Island; Pres B. H. Patterson, Baxley; Rec. Sec. W. L. Smith, Hawkinsville; Cor. Sec. L. D. Carpenter, Atlanta.

*Illinois State Dental Society*—Meets annually. Pres. W. T. Magill, Rock Island; Sec. J. W. Wassal, Chicago. Next place of meeting, Jacksonville, second Tuesday in May, 1888.

*Indiana State Dental Society*—Meets next in Terre Haute on the last Tuesday of June, 1888. Pres. W. W. Wilson, Richmond; Sec. Robert W. Van Valzah, Terre Haute.

*Iowa State Dental Society*—Meets annually. Next meeting in Iowa City, on the first Tuesday in May, 1888. Pres. W. P. Dickinson, Dubuque; Rec. Sec. J. B. Montfort, Fairfield.

*Kentucky State Dental Association*—Meets annually, first Tuesday in June, 1888. Next meeting in Louisville. Pres. W. S. Smith, Newcastle; Sec. E. C. Dunn, Louisville.

*Kansas State Dental Association*—Pres. R. E. Nickles, Selma; Sec. C. B. Gunn, Leavenworth. Next meeting will be held at Topeka, April 24th, 1888.

*Maine Dental Society*—Meets in August, at Thomaston. Pres. E. J. Robert, Augusta, Sec. C. E. Hussey, Beddeford.

*Maryland State Dental Association*—Meets annually next November 13. Pres. D. F. Pennington; Rec. Sec. Wm. A. Mills; Cor, Sec. S. C. Pennington

*Massachusetts Dental Society*—Meets annually in Boston, second Thursday in December. Pres. S. G. Stevens, Boston; Rec. and Cor. Sec. G. F. Eames, Boston.

*Michigan State Dental Association*—Meets annually. Next meeting at Ann Arbor, last Tuesday in March, 1888. Pres. J. A. Robinson, Jackson; Sec. J. B. McGregor, Port Huron; Treas. H. K. Lathrop, Detroit.

*Mississippi State Dental Association*—Will meet August 4, 1887, at Jackson. Pres. Morgan Adams, Sardis; Sec. A. N. Helzim, Jackson.

*Minnesota Dental Association*—Meets annually second Wednesday in July, 1888, at St. Paul. Pres. H. L. Cruttenden, Northfield; Sec. D. W. Edwards, Le Sueur.

*Missouri Dental Association*—Meets annually on July 10th, 1888. Next meeting at Pertel Springs. Pres. Wm. N. Morrison, St. Louis; Sec. John G. Harper, St. Louis.

*Nebraska State Dental Society*—Meets annually. Next meeting third Tuesday in May 1888, at Beatrice; Pres. J. J. Willey, Wahoo; Sec. C. R. Tefft, Lincoln.

*New Jersey State Dental Society*—Meets annually. Next meeting at Cape May, on Wednesday, July, 20th, 1888. Pres. G. C. Brown, Elizabeth; Sec. C. A. Meeker, Newark.

*New Hampshire Dental Society*—Meets in Concord on the third Tuesday of June, 1888. Pres. C. B. Russell, Farmington; Sec. E. B. Davis.

*North Carolina State Dental Society*—Meets annually. Next meeting at Raleigh, last Tuesday in May, 1888. Pres. Thos. M. Hunter, Fayetteville; Sec. H. C. Hearing, Concord.

*Ohio State Dental Society*—Meets annually. Next meeting at Springfield, last Tuesday of October, 1887. Pres. H. H. Harrison, Cadiz; Sec. J. R. Callahan, Hillsboro.

*Pennsylvania State Dental Society*—Meets second Tuesday of September, 1888, at Philadelphia; Pres. W. H. Roop, Philadelphia; Rec. and Cor. Sec. Th. F. Chupein, Philadelphia.

*Rhode Island Dental Association*—Pres. F. G. Eddy, Providence; Sec. L. L. Buckland, Providence. Meets quarterly, first Tuesday in July, October, January, and April. Annual Meeting first Tuesday in July.

*South Carolina State Dental Association*—Meets annually. Pres. L. S. Wolfe, Orangburg; Sec. E. C. Bigell, Batesburg; Rec. Cor. Sec. R. A. Smith, Charleston. Next meeting at Greenville, third Tuesday in July, 1888.

*Texas State Dental Association*—Meets in the city of Dallas, May 1st, 1888. Pres. R. E. Grant, Austin; Sec. G. M. Patten, Huntsville.

*Tennessee Dental Association*—Meets Annually. Next meeting at Memphis, first Tuesday in July 1888. Pres. Gordon White, Nashville; Rec. Sec. D. R. Stubelfield, Nashville; Cor. Sec. H. E. Beach, Clarksville.

*The Dental Society of the State of New York*—Meets annually on the second Wednesday in May. Next session at Albany, May 9, 1888. Pres. Norman W. Kingsley, New York; Rec. Sec. J. Ewd. Line, Rochester; Cor. Sec. W. H. Atkinson, New York.

*Vermont State Dental Society*—Meets annually. Next meeting at Burlington, March 16, 1888. Pres. J. P. Parker; Sec. Thos. Mound, Rutland.

*Virginia State Dental Association*—Meets in Charlottsville, Tuesday, August 19, 1886. Pres. J. Hall Moore; Sec. L. M. Cowarden, Richmond.

*Wisconsin State Dental Society*—Meets annually. Next meeting at Milwaukee, July 20, 1888. Pres. B. G. Marklein, Milwaukee; Sec. C. A. Southwell, Appleton, Wis.

### LOCAL SOCIETIES.

*American Academy of Dental Science*—Meets annually in Boston, Mass., on the first Wednesday in October, 1887. Pres. J. H. Batchelder; Rec. Sec. E. E. Hopkins; Cor. Sec. E. B. Hitchcock, Boston.

*Brooklyn Dental Society and Second District Society United*—Meets quarterly—Pres. T. H. Holly; Rec. Sec. A. N. Russell, Brooklyn.

*Central Dental Association of Northern New Jersey*—Meets annually in February. Pres. C. A. Meeker, Newark, Sec. G. Carleton Brown, Elizabeth.

*Central Illinois Dental Society*—Meets annually. Next meeting at Springfield, Oct. 11 and 12, 1887; Pres. E. B. Call, Peoria; Sec. W. A. Johnston, Peoria.

*Chicago Dental Society*—Meets monthly. Pres. F. H. Gardiner; Sec. J. G. Reid.

*Chicago Dental Club*—Meets monthly. Pres. L. P. Haskell; Sec. Arthur B. Freeman.

*Connecticut Valley Dental Society*—Meets 27 and 28 of Oct. 1887, at Savin Rock Conn. Pres. C. T. Stockwell, Springfield, Mass. Sec. A. M. Ross, Chicopee.

*Detroit Dental Society*—Meets monthly, Pres. Geo. S. Shattuck.

*Eighth District Dental Society, N. Y.*—Meets semi-annually. Next meetlng in Buffalo, April 15, 1887. Pres. S. A. Freeman; Sec. C. S. Butler, Buffalo.

*East Tennessee Dental Association*—Meets annually. Next meeting will be held at Chattanooga, Tenn., on the first Tuesday of Aug., 1888. Pres. R. A. B. Noyers, Sec. S. N. Prothro.

*Mad River Dental Society*—Meets annually. Next meeting at Dayton, Tuesday, May 19, 1888. Pres. C. Bradley, Dayton; Sec. and Treas. L. C. Adams, Dayton,

*Mississippi Valley Dental Society*—Meets annually at Cincinnati, on first Wednesday in March, 1888. Pres. A. W. Harlan, Chicago, Ill.; Sec. A. G. Rose, Cincinnati.

*New England Dental Society*—Meets annually. Time and place of next meeting is to be decided by the Ex Com. Pres., A. M. Dudley, Salem, Mass.; Sec. A. H. Gilson, Boston, Mass.

*Northern Ohio Dental Association*—Meets annually. Next meeting at Painesville, O. on the second Tuesday in May, 1888. Pres. Geo. H. Wilson, Painesville; Rec. Sec. W. H. Whitsler, Youngstown; Cor. Sec. S. B. Dewey, Cleveland, O.

*Northwestern Dental Association* (embraces portions of Dakota, Minnesota and Manitoba) meets annually. Next meeting at Fargo, Dakota, fourth Tuesday in July, 1888. Pres. J. W. Cloes, Jamestown, Dak. Sec. S. J. Hill, Fargo, Dak.

*Ohio Dental College Association*—Meets annually, Tuesday, March 2, 1888, at Cincinnati. Pres. H. M. Reid, Minneapolis; Rec. Sec. F. A. Hunter, Cincinnati.

*Odontological Society of Pennsylvania*—Meets on the first Saturday evening of each month, at 8 o'clock p. m., in the offices of the members; Pres. E. T. Darby, Rec. Sec. Ambler Tees.

*Odontological Society of New York City*—Meets the third Tuesday of each month. Pres. Wm. Jarvie; Rec. Sec. E. T. Payne.

*Odontological Society of Cincinnati*—Meets monthly. Pres. A. Berry. Sec. N. S. Hoff.

*Pennsylvania Association of Dental Surgeons*—Pres. J. H. Githens. Rec. Sec. Theo. F. Chupein.

*Pittsburg Dental Society*—Meets the last Tuesday Evening of each month. Pres. J. B. Williams, Homestead; Sec. N. L. Reinecke.

*Fifth District Dental Society of N. Y.*—Meets semi-annually. Next meeting in Birmingham, in October. Pres. C. C. Smith, Ilion; Sec. C. J. Peters, Syracuse.

*San Francisco Dental Society*—Meets the second Monday evening of each month. Pres. W. J. Younger; Sec. W. A. Knowles; Treas. J. J. Birge.

*Sixth District Dental Society of N. Y.*—Meets semi-annually. Next meeting at Cortland, Thursday, Oct. 6th, 1888. Pres. M. D. Jewett, Richford Springs; Sec. E. D. Downs, Owego.

*St. Louis Dental Society*—Meets first Tuesday in each month. Pres. Henry Fisher; Cor. Sec. W. N. Morrison; Rec. Sec. John G. Harper.

*Washington Dental Society*—Meets second Tuesday of each month. Pres. R. Finley Hunt; Rec. Sec. H. M. Schooly.

*Southern Dental Association*—Meets in Louisville, Ky., on the 1st Tuesday of August, 1888. Pres. B. H. Catching, Atlanta, Ga.; Cor. Sec. J. Y. Crawford, Nashville, Tenn.; Sec. L. P. Datterer, Charleston, S. C.

*First District Dental Society of the State of New York*—Meets annually. Pres. A. L. Northrop; Sec. J. E. Dexter, New York.

---

### EXCHANGES.

St. Louis, Medical and Surgical Journal.
The Pacific Medical and Surgical Journal, San Francisco.
Cincinnati Lancet and Clinic.
Dental Cosmos, Philadelphia.
Chicago Medical Examiner.
The Detroit Review of Medicine and Pharmacy.
American Journal of Dental Science, Baltimore.
Indiana Medical Journal.
Archives of Dentistry.
The Sanitarian, New York City.
L'art Dentaire, Paris.
Le Progres Dentare, Paris.
Deutsche Monatsschrift fuer Zahnheilkunde Leipzig.—Arthur Felix.
Physician and Surgeon, Ann Arbor, Mich.
Ohio State Journal of Dental Science, Xenia, Ohio
The Microscope, Ann Arbor, Mich.
Independent Practitioner, N. Y.
The Dental Record, London.
The Journal of the British Dental Association, London.
The Southern Dental Journal.
Items of Interest, Philadelphia.
The Dental Practitioner.—The Cincinnati "Medical" News.
The British Journal of Dental Science, London, Eng.
Facts.
The Dental Advertiser.
The American Practitioner.
The Messenger of Dentistry (Russian).
The Louisville Medical News.
The Medical World.
American Journal of Obstetrics.
The American Pharmacist.
The Nashville Journal of Medicine and Surgery.
Science.
Medical and Surgical Reporter, Philadelphia, Pa.
Dental Headlight.
Cincinnati Medical and Dental Journal.
The New York Medical and Surgical Journal.
The Dental Review.

### LOUISVILLE

### COLLEGE OF DENTISTRY,

*Dental Department of Central University,*

### LOUISVILLE, KY.

THE REGULAR SESSION OF THIS INSTITUTION BEGINS JANUARY 20, 1887, AND ENDS JUNE 17, 1887.

### FACULTY.

A. WILKES SMITH, M. D., D. D. S., Professor of Oral and Dental Surgery and Operative Dentistry. Dean of Faculty.

CHARLES G. EDWARDS, D. D. S., Professor of Prosthetic and Clinical Dentistry.

A. M. CARTLEDGE, M. D., Professor of Surgery.

DUDLEY S. REYNOLDS, M. D., Professor of Pathology and Hygiene.

FRANK C. WILSON, M. D., Professor of Principles and Practice of Medicine.

SAMUEL G. DABNEY, M. D., Professor or Physiology and Histology.

JOHN A. LARRABEE, M. D., Professor of Materia Medica and Therapeutics.

C. SKINNER, M. D., Professor of Anatomy.

JAS. LEWIS HOWE, M. D., Ph. D., F. C. S., Professor of Medical Chemistry and Toxicology. Scientist to the Polytechnic Society of Kentucky.

### DEMONSTRATORS.

H. B. TILESTON, D. D. S., Demonstrator of Operative Dentistry.

JOHN H. BALDWIN, D. D. S., Demonstrator of Prosthetic Dentistry.

GEORGE F. MARTIN, M. D., Demonstrator of Anatomy.

### FEES.

| | |
|---|---|
| Matriculation, | $5 00 |
| Demonstrations in Anatomy, | 10 00 |
| Lectures, | 75 00 |
| Graduation, | 30 00 |
| Hospital Ticket (required by city), | 5 00 |

### THE DENTAL COLLEGE

OF THE

### University of Michigan.

The Eleventh Annual Session of this Institution will commence on the 1st of October and close on the last Thursday of June, thus making a course of nine months. The regular course of instruction commences at once, and will continue through the term, with the customary vacation.

The students in this department will receive instruction in Anatomy, Physiology, Pathology, Chemistry, Materia Medica, Therapeutics, and Surgery, from the Professors of their respective branches in the *Department of Medicine and Surgery* of the University, where lectures commence, and continue the same as with the Dental College. There will also, in addition, be a special course upon Oral Pathology and Surgery, which will embrace from thirty to thirty-five lectures.

### FACULTY OF THE DENTAL DEPARTMENT.

| | |
|---|---|
| J. B. ANGELL, LL.D. | President. |
| J. TAFT, M.D., D.D.S. | Principles and Practice of Operative Dentistry. |
| CORYDON L. FORD, M.D., D.D.S. | Anatomy and Physiology. |
| J. A. WATLING, D.D.S. | Clinical and Mechanical Dentistry. |
| W. H. DORRANCE, DD.S. | Prosthetic Dentistry. |
| J. N. Martin, M.D., | Lecturer on Oral Pathology and Surgery. |
| MARGARET HUMPHREYS, D.D.S. | Demonstrator of Clinical Dentistry. |
| ———————— | Demonstrator of Prosthetic Dentistry. |

Students should be promptly present at the College on Wednesday, September 30th, at 10 o'clock, A.M., to make the preliminary arrangements for entering upon regular work. Seats in the lecture room are assigned by election to students in the order of registration on the Steward's Books; and each student is expected to occupy during the session such seat as he may select. Students, on arriving in Ann Arbor, should call at the Steward's office.

### CONDITIONS OF GRADUATION.

The candidate must be twenty-one years of age. *He must furnish satisfactory evidence of good moral character.*

He must devote three years to the study of his profession. He must attend two full courses of lectures in the Dental College, or one in some college having an equal standard of requirements, and the last one here, and we recommend that he attend three courses regularly.

He must sustain an examination satisfactory to the Faculty in all the branches taught.

A graduate of the Medical College may enter the Senior Class, and, if found qualified, may graduate after two years have been devoted to the study of dentistry.

### FEES AND EXPENSES.

The fees, which must be paid in advance, are as follows:

RESIDENTS OF MICHIGAN.—Matriculation fee, $10.00; annual dues, $25.00

NON-RESIDENTS.—Matriculation, $25.00; annual dues, $35.00.

GRADUATION FEE.—For all alike, $10.00. The admission fee is paid but once, and entitles the student to the privileges of permanent membership in any department of the University. The annual dues are paid the first year and every year thereafter while at the University.

For further particulars, address the Dean of the Dental College, Ann Arbor, Michigan.

**J. TAFT, Dean.**

Jan-86-1y

### LOUISVILLE

### COLLEGE OF DENTISTRY,

*Dental Department of Central University,*

**LOUISVILLE, KY.**

THE REGULAR SESSION OF THIS INSTITUTION BEGINS JANUARY 20, 1887, AND ENDS JUNE 17, 1887.

---

### FACULTY.

A. WILKES SMITH, M.D., D.D.S., Professor of Oral and Dental Surgery and Operative Dentistry. Dean of Faculty.

CHARLES G EDWARDS, D. D. S., Professor of Prosthetic and Clinical Dentistry.

A. M. CARTLEDGE, M.D., Professor of Surgery.

DUDLEY S. REYNOLDS, M.D., Professor of Pathology and Hygiene.

FRANK C. WILSON, M.D., Professor of Principles and Practice of Medicine.

SAMUEL G. DABNEY, M.D., Professor or Physiology and Histology.

JOHN A. LARRABEE, M.D., Professor of Materia Medica and Therapeutics.

C. SKINNER, M.D., Professor of Anatomy.

JAS. LEWIS HOWE, M.D., Ph.D., F.C.S., Professor of Medical Chemistry and Toxicology. Scientist to the Polytechnic Society of Kentucky.

---

### DEMONSTRATORS.

H. B. TILESTON, D.D.S., Demonstrator of Operative Dentistry.

JOHN H. BALDWIN, D.D.S., Demonstrator of Prosthetic Dentistry.

GEORGE F. MARTIN, M.D., Demonstrator of Anatomy.

---

### FEES.

| | |
|---|---|
| Matriculation, | $5 00 |
| Demonstrations in Anatomy, | 10 00 |
| Lectures, | 75 00 |
| Graduation, | 30 00 |
| Hospital Ticket (required by city), | 5 00 |

### Northwestern College of Dental Surgery

**SOUTHEAST COR. WABASH AV. AND TWELFTH STREET,**

CHICAGO, ILL.

**BOARD OF DIRECTORS.**

H. C. MAGNUSSON, President.
N. J. ROBERTS,
F. H. B. McDOWELL, Secretary.

**BOARD OF EDUCATIONAL CONTROL.**

J. E. HEQUEMBOURG, M.D., President. A. P. MERRILL, D.D.S.
JOSEPH HAVEN, M.D. N. J. ROBERTS, D.D.S.
BYRON D. PALMER, D.D.S.

**SUPERVISORS OF THE CLINIC.**

M. Stout, D.D.S. A. P. Merrill, D.D.S. Byron D. Palmer, D.D.S.

**FACULTY.**

G. C. PAOLI, A.M., M.D., Emeritus Professor of Materia Medica.
N. P. PEARSON, A.M., M.D., Emeritus Professor of Pathology.

JOSEPH HAVEN, M.D., Professor of Physiology and Dean of the Faculty.
A. P. MERRILL, D.D.S., Professor of Operative Dentistry & Dental Histology.
BYRON D. PALMER, D.D.S. Professor of Prosthetic Dentistry.
M. STOUT, D.D.S., Professor of Clinlcal Dentistry.
NORMAN J. ROBERTS, D.D.S., Professor of Oral Surgery.
J. E. HEQUEMBOURG, M.D., Professor of Anatomy and Principles and Practice of Surgery and Surgical Pathology.
F. C. CALDWELL, M.D., Professor of Materia Medica.
J. H. SALISBURY, M.D., Professor of Chemistry.
J. H. LYON, M.D., Professor of Dental Pathology.

**DEMONSTRATORS.**

T. C. RIVERA, M.D., - - - Demonstrator of Chemistry.
Dr. G. F. SCHAFER, Jr., - Demonstrator of Mechanical Dentistry.
Dr. L. E. IRELAND, - - Demonstrator of Operative Dentistry.

**Announcement for the Course of 1887–8.**

On Tuesday, October 4, 1887, the Northwestern College of Dental Surgery commences its Third Annual Session; and, in the interest of advanced education in the Dental Profession, the Directors have decided to extend the school year from the present course of six months to one of nine months, thus giving a longer time for study and practical instruction and avoiding the crowding necessary in a shorter term.

From its first foundation this College has succeeded far beyond the hopes of its founders and the most sanguine expectations of its friends, and the number and character of its students have exceeded all anticipations. While we feel justified in saying that from its organization the facilities and opportunities for securing a dental education in it have been equal to any other similar institution of learning in the country, we are equally aware that the profession and the people would justly have more confidence in the scholarship and capabilities of graduates of a dental college that requires two terms of nine months' each, than they would have in the graduates of a college that requires two terms of but four or five months' attendance at most, with a preliminary term of a month or two, the attendance upon which is optional with the student. The motive for this change is one only of professional and public interest; but we expect it will meet with the commendation of both. The outlines of the course of instruction, which is a graded one so arranged as to take the student step by step through all the branches of medical and dental theory and practical work to a high standard of profession skill, will be found in the annual announcement, which will be furnished upon application.

The clinics of this College—owing to its favorable location, and the high reputation attained by its clinical professors and demonstrators in the treatment of oral diseases, and the minor operations in dentistry—are the most largely attended of any of the dental colleges of the country; and the infinite variety of operations which a population of over 700,000 offers, renders the experience gained in the clinic rooms of this College invaluable to the student and practitioner.

Every candidate for admission must be eighteen years of age, and must present to the Faculty satisfactory evidence of a good moral character. Unless already a matriculate of the College, or of some other recognized college or university, or a graduate of some recognized academy or high school, or holding a teacher's certificate, he must pass an examination in Arithmetic, Geography, English Grammer, and Composition, United States History, and Natural Philosophy. (After July 1, 1887, in Elementary Chemistry.) He shall subscribe to Article II., Section 3, of the Code of Ethics of the American Dental Association.

The candidate for the degree of Doctor of Dental Surgery must have attained the age of twenty one years. He shall have passed a satisfactory examination, both oral and written—a written examination being substituted in this College for a thesis. He shall have studied dentistry three years, including two courses of lectures, one of which shall be at this institution. Graduates in medicine may apply for the degree of D.D.S., after having had two full years of practical instruction or experience in dentistry, one year of which, including one course of lectures, must be spent in the College. After these requirements have been complied with, upon recommendation of the Faculty and approval by the Board of Directors, the candidate will receive the degree of Doctor of Dental Surgery.

### FEES.

For the first year a student is a member of the college the fee is $100, payable in two installments of $75 and $25; for the second year $100, in payments of $60 and $40; for any subsequent year, $50. There are no other fees, either for matriculation, demonstration, or diploma, the above covering all the tuition and graduation fees of the complete course. For information, address

F. H. B. McDOWELL, *Secretary*,
1201 Wabash Avenue, Chicago, Ill.

### THE DENTAL COLLEGE

OF THE

### University of Michigan.

The Eleventh Annual Session of this Institution will commence on the 1st of October and close on the last Thursday of June, thus making a course of nine months. The regular course of instruction commences at once, and will continue through the term, with the customary vacation.

The students in this department will receive instruction in Anatomy, Physiology, Pathology, Chemistry, Materia Medica, Therapeutics, and Surgery, from the Professors of their respective branches in the *Department of Medicine and Surgery* of the University, where lectures commence, and continue the same as with the Dental College. There will also, in addition, be a special course upon Oral Pathology and Surgery, which will embrace from thirty to thirty-five lectures.

### FACULTY OF THE DENTAL DEPARTMENT.

| | |
|---|---|
| J. B. ANGELL, LL.D. | President. |
| J. TAFT, M.D., D.D.S. | Principles and Practice of Operative Dentistry. |
| CORYDON L. FORD, M.D., D.D.S. | Anatomy and Physiology. |
| J. A. WATLING, D.D.S. | Clinical and Mechanical Dentistry. |
| W. H. DORRANCE, DD.S. | Prosthetic Dentistry. |
| J. N. Martin, M.D., | Lecturer on Oral Pathology and Surgery. |
| MARGARET HUMPHREYS, D.D.S. | Demonstrator of Clinical Dentistry. |
| ——————— | Demonstrator of Prosthetic Dentistry. |

Students should be promptly present at the College on Wednesday, September 30th, at 10 o'clock, A.M., to make the preliminary arrangements for entering upon regular work. Seats in the lecture room are assigned by election to students in the order of registration on the Steward's Books; and each student is expected to occupy during the session such seat as he may select. Students, on arriving in Ann Arbor, should call at the Steward's office.

### CONDITIONS OF GRADUATION.

The candidate must be twenty-one years of age. *He must furnish satisfactory evidence of good moral character.*

He must devote three years to the study of his profession. He must attend two full courses of lectures in the Dental College, or one in some college having an equal standard of requirements, and the last one here, and we recommend that he attend three courses regularly.

He must sustain an examination satisfactory to the Faculty in all the branches taught.

A graduate of the Medical College may enter the Senior Class, and, if found qualified, may graduate after two years have been devoted to the study of dentistry.

### FEES AND EXPENSES.

The fees, which must be paid in advance, are as follows:

RESIDENTS OF MICHIGAN.—Matriculation fee, $10.00; annual dues, $25.00

NON-RESIDENTS.—Matriculation, $25.00; annual dues, $35.00.

GRADUATION FEE.—For all alike, $10.00. The admission fee is paid but once, and entitles the student to the privileges of permanent membership in any department of the University. The annual dues are paid the first year and every year thereafter while at the University.

For further particulars, address the Dean of the Dental College, Ann Arbor, Michigan.

**J. TAFT, Dean.**

Jan-86-1y

### LOUISVILLE

### COLLEGE OF DENTISTRY,

*Dental Department of Central University,*

**LOUISVILLE, KY.**

**THE REGULAR SESSION OF THIS INSTITUTION BEGINS JANUARY 20, 1887, AND ENDS JUNE 17, 1887.**

### FACULTY.

A. WILKES SMITH, M.D., D.D.S., Professor of Oral and Dental Surgery and Operative Dentistry. Dean of Faculty.

CHARLES G. EDWARDS, D. D. S., Professor of Prosthetic and Clinical Dentistry.

A. M. CARTLEDGE, M.D., Professor of Surgery.

DUDLEY S. REYNOLDS, M.D., Professor of Pathology and Hygiene.

FRANK C. WILSON, M. D., Professor of Principles and Practice of Medicine.

SAMUEL G. DABNEY, M. D., Professor or Physiology and Histology.

JOHN A. LARRABEE, M. D., Professor of Materia Medica and Therapeutics.

C. SKINNER, M. D., Professor of Anatomy.

JAS. LEWIS HOWE, M.D., Ph.D., F.C.S., Professor of Medical Chemistry and Toxicology. Scientist to the Polytechnic Society of Kentucky.

### DEMONSTRATORS.

H. B. TILESTON, D.D.S., Demonstrator of Operative Dentistry.

JOHN H. BALDWIN, D.D.S., Demonstrator of Prosthetic Dentistry.

GEORGE F. MARTIN, M.D., Demonstrator of Anatomy.

### FEES.

| | |
|---|---|
| Matriculation, | $5 00 |
| Demonstrations in Anatomy, | 10 00 |
| Lectures, | 75 00 |
| Graduation, | 30 00 |
| Hospital Ticket (required by city), | 5 00 |

### Northwestern College of Dental Surgery

**SOUTHEAST COR. WABASH AV. AND TWELFTH STREET,**

CHICAGO, ILL.

### BOARD OF DIRECTORS.

H. C. MAGNUSSON, President.
N. J. ROBERTS,
F. H. B. McDOWELL, Secretary.

### BOARD OF EDUCATIONAL CONTROL.

J. E. HEQUEMBOURG, M.D., President.
A. P. MERRILL, D.D.S.
JOSEPH HAVEN, M.D.
N. J. ROBERTS, D.D.S.
BYRON D. PALMER, D.D.S.

### SUPERVISORS OF THE CLINIC.

M. STOUT, D.D.S. A. P. MERRILL, D.D.S. BYRON D. PALMER, D.D.S.

### FACULTY.

G. C. PAOLI, A.M., M.D., Emeritus Professor of Materia Medica.
N. P. PEARSON, A.M., M.D., Emeritus Professor of Pathology.

JOSEPH HAVEN, M.D., Professor of Physiology and Dean of the Faculty.
A.P. MERRILL, D.D.S., Professor of Operative Dentistry & Dental Histology.
BYRON D. PALMER, D.D.S. Professor of Prosthetic Dentistry.
M. STOUT, D.D.S., Professor of Clinlcal Dentistry.
NORMAN J. ROBERTS, D.D.S., Professor of Oral Surgery.
J. E. HEQUEMBOURG, M.D., Professor of Anatomy and Principles and Practice of Surgery and Surgical Pathology.
F. C. CALDWELL, M.D., Professor of Materia Medica.
J. H. SALISBURY, M.D., Professor of Chemistry.
J. H. LYON, M.D., Professor of Dental Pathology.

### DEMONSTRATORS.

T. C. RIVERA, M.D., - - - Demonstrator of Chemistry.
DR. G. F. SCHAFER, JR., - Demonstrator of Mechanical Dentistry.
DR. L. E. IRELAND, - - Demonstrator of Operative Dentistry.

### Announcement for the Course of 1887-8.

On Tuesday, October 4, 1887, the Northwestern College of Dental Surgery commences its Third Annual Session; and, in the interest of advanced education in the Dental Profession, the Directors have decided to extend the school year from the present course of six months to one of nine months, thus giving a longer time for study and practical instruction and avoiding the crowding necessary in a shorter term.

From its first foundation this College has succeeded far beyond the hopes of its founders and the most sanguine expectations of its friends, and the number and character of its students have exceeded all anticipations. While we feel justified in saying that from its organization the facilities and opportunities for securing a dental education in it have been equal to any other similar institution of learning in the country, we are equally aware that the profession and the people would justly have more confidence in the scholarship and capabilities of graduates of a dental college that requires two terms of nine months' each, than they would have in the graduates of a college that requires two terms of but four or five months' attendance at most, with a preliminary term of a month or two, the attendance upon which is optional with the student. The motive for this change is one only of professional and public interest; but we expect it will meet with the commendation of both. The outlines of the course of instruction, which is a graded one so arranged as to take the student step by step through all the branches of medical and dental theory and practical work to a high standard of profession skill, will be found in the annual announcement, which will be furnished upon application.

The clinics of this College—owing to its favorable location, and the high reputation attained by its clinical professors and demonstrators in the treatment of oral diseases, and the minor operations in dentistry—are the most largely attended of any of the dental colleges of the country; and the infinite variety of operations which a population of over 700,000 offers, renders the experience gained in the clinic rooms of this College invaluable to the student and practitioner.

Every candidate for admission must be eighteen years of age, and must present to the Faculty satisfactory evidence of a good moral character. Unless already a matriculate of the College, or of some other recognized college or university, or a graduate of some recognized academy or high school, or holding a teacher's certificate, he must pass an examination in Arithmetic, Geography, English Grammer, and Composition, United States History, and Natural Philosophy. (After July 1, 1887, in Elementary Chemistry.) He shall subscribe to Article II., Section 3, of the Code of Ethics of the American Dental Association.

The candidate for the degree of Doctor of Dental Surgery must have attained the age of twenty one years. He shall have passed a satisfactory examination, both oral and written—a written examination being substituted in this College for a thesis. He shall have studied dentistry three years, including two courses of lectures, one of which shall be at this institution. Graduates in medicine may apply for the degree of D.D.S., after having had two full years of practical instruction or experience in dentistry, one year of which, including one course of lectures, must be spent in the College. After these requirements have been complied with, upon recommendation of the Faculty and approval by the Board of Directors, the candidate will receive the degree of Doctor of Dental Surgery.

---

### FEES.

For the first year a student is a member of the college the fee is $100, payable in two installments of $75 and $25; for the second year $100, in payments of $60 and $40; for any subsequent year, $50. There are no other fees, either for matriculation, demonstration, or diploma, the above covering all the tuition and graduation fees of the complete course. For information, address

F. H. B. McDOWELL, *Secretary*,

1201 WABASH AVENUE, CHICAGO, ILL.

### THE DENTAL COLLEGE

OF THE

### University of Michigan.

The Eleventh Annual Session of this Institution will commence on the 1st of October and close on the last Thursday of June, thus making a course of nine months. The regular course of instruction commences at once, and will continue through the term, with the customary vacation.

The students in this department will receive instruction in Anatomy, Physiology, Pathology, Chemistry, Materia Medica, Therapeutics, and Surgery, from the Professors of their respective branches in the *Department of Medicine and Surgery* of the University, where lectures commence, and continue the same as with the Dental College. There will also, in addition, be a special course upon Oral Pathology and Surgery, which will embrace from thirty to thirty-five lectures.

### FACULTY OF THE DENTAL DEPARTMENT.

| | |
|---|---|
| J. B. ANGELL, LL.D. | President. |
| J. TAFT, M.D., D.D.S. | Principles and Practice of Operative Dentistry. |
| CORYDON L. FORD, M.D., D.D.S. | Anatomy and Physiology. |
| J. A. WATLING, D.D.S. | Clinical and Mechanical Dentistry. |
| W. H. DORRANCE, DD.S. | Prosthetic Dentistry. |
| J. N. Martin, M.D., | Lecturer on Oral Pathology and Surgery. |
| MARGARET HUMPHREYS, D.D.S. | Demonstrator of Clinical Dentistry. |
| ———————— | Demonstrator of Prosthetic Dentistry. |

Students should be promptly present at the College on Wednesday, September 30th, at 10 o'clock, A.M., to make the preliminary arrangements for entering upon regular work. Seats in the lecture room are assigned by election to students in the order of registration on the Steward's Books; and each student is expected to occupy during the session such seat as he may select. Students, on arriving in Ann Arbor, should call at the Steward's office.

### CONDITIONS OF GRADUATION.

The candidate must be twenty-one years of age. *He must furnish satisfactory evidence of good moral character.*

He must devote three years to the study of his profession. He must attend two full courses of lectures in the Dental College, or one in some college having an equal standard of requirements, and the last one here, and we recommend that he attend three courses regularly.

He must sustain an examination satisfactory to the Faculty in all the branches taught.

A graduate of the Medical College may enter the Senior Class, and, if found qualified, may graduate after two years have been devoted to the study of dentistry.

### FEES AND EXPENSES.

The fees, which must be paid in advance, are as follows:

Residents of Michigan.—Matriculation fee, $10.00; annual dues, $25.00

Non-Residents.—Matriculation, $25.00; annual dues, $35.00.

Graduation Fee.—For all alike, $10.00. The admission fee is paid but once, and entitles the student to the privileges of permanent membership in any department of the University. The annual dues are paid the first year and every year thereafter while at the University.

For further particulars, address the Dean of the Dental College, Ann Arbor, Michigan.

**J. TAFT, Dean.**

Jan-86-1y

### LOUISVILLE
### COLLEGE OF DENTISTRY,

*Dental Department of Central University,*

**LOUISVILLE, KY.**

**THE REGULAR SESSION OF THIS INSTITUTION BEGINS JANUARY 20, 1887, AND ENDS JUNE 17, 1887.**

### FACULTY.

A. WILKES SMITH, M.D., D.D.S., Professor of Oral and Dental Surgery and Operative Dentistry. Dean of Faculty.

CHARLES G. EDWARDS, D. D. S., Professor of Prosthetic and Clinical Dentistry.

A. M. CARTLEDGE, M.D., Professor of Surgery.

DUDLEY S. REYNOLDS, M.D., Professor of Pathology and Hygiene.

FRANK C. WILSON, M. D., Professor of Principles and Practice of Medicine.

SAMUEL G. DABNEY, M. D., Professor or Physiology and Histology.

JOHN A. LARRABEE, M.D., Professor of Materia Medica and Therapeutics.

C. SKINNER, M.D., Professor of Anatomy.

JAS. LEWIS HOWE, M.D., Ph.D., F.C.S., Professor of Medical Chemistry and Toxicology. Scientist to the Polytechnic Society of Kentucky.

### DEMONSTRATORS.

H. B. TILESTON, D.D.S., Demonstrator of Operative Dentistry.

JOHN H. BALDWIN, D.D.S., Demonstrator of Prosthetic Dentistry.

GEORGE F. MARTIN, M.D., Demonstrator of Anatomy.

### FEES.

| | |
|---|---|
| Matriculation, | $5 00 |
| Demonstrations in Anatomy, | 10 00 |
| Lectures, | 75 00 |
| Graduation, | 30 00 |
| Hospital Ticket (required by city), | 5 00 |

1845. ★ 1887.

### Ohio College of Dental Surgery.

**CINCINNATI, OHIO.**

SESSION 1887-8.

### FACULTY.

J. S. CASSIDY, M.D., D.D.S., *Professor of Chemistry and Materia Medica.*
H. A. SMITH, D.D.S., DEAN OF THE FACULTY, *Professor of Operative Dentistry and Dental Pathology.*
C. M. WRIGHT, D.D.S., *Professor of Physiology and General Pathology.*
WM. KNIGHT, M.D., *Professor of Anatomy and Oral Surgery.*
GRANT MOLLYNEAUX, D.D.S., *Professor of Mechanical Dentistry and Metallurgy.*

GEO. W. KEELY, D.D.S., *Lecturer on Irregularities of the Teeth.*
OTTO ARNOLD, D.D.S., *Lecturer on Anæsthetics.*

### DEMONSTRATORS.

A. T. OLMSTED, D.D.S., *Demonstrator of Operative Dentistry.*
B. C. HINKLEY, D.D.S., *Ass't Demonstrator of Operative Dentistry.*
JAS. SILCOTT, D.D.S., *Demonstrator of Mechanical Dentistry.*
G. S. JUNKERMAN, M.D., D.D.S., *Demonstrator of Analytical Chemistry.*
H. C. MATLACK, D.D.S., *Demonstrator of Anatomy.*
C. H. MARTIN, D.D.S., *Assistant to Department of Orthodontia.*

The Forty-Second Annual Winter Session begins Tuesday, October 4, 1887, and continues uninterruptedly through five months.

At the close of the regular Session a Spring Course of practical instruction will be inaugurated.

Requirements for Admission and Graduation are those adopted by the National Association of Dental Faculties.

### TUITION FEES:

| | |
|---|---|
| Matriculation Fee (but once) | $ 5 00 |
| Professors' Tickets for first session | 75 00 |
| Professors' Tickets for second year | 50 00 |
| Dissecting Ticket, including material | 10 00 |
| Analytical Chemistry | 10 00 |
| Diploma Fee | 25 00 |

**For further information and announcement, address,**

**H. A. SMITH, DEAN,**
**128 Garfield Place, Cincinnati, O.**

NOTICE.—Parties wishing polishing strips cut to order can have it done by sending us their order and sample of grade and width, without extra charge. See advertisement on page 6.

SAM'L A. CROCKER & CO.

FOR SALE CHEAP.—Dental practice, office fixtures, &c., in a rapidly growing town of six thousand inhabitants, in the blue grass district of Kentucky. An excellent opportunity for a good dentist. Satisfactory reasons for desiring to sell. Address, J. W. S.

CARE SAM'L A. CROCKER & CO.

### MITCHELL'S DENTAL THAPSIA PLASTERS.

These plasters are an indispensable article of comfort for all afflicted with the excrutiating pain of toothache, or diseased gums. They are readily adapted to the parts afflicted, and by the immediate relief afforded recommend themselves to all sufferers.

They do not crack, nor peel off.

DIRECTIONS.—Press the plaster to the gum until it adheres. Irritation will begin at once, and last for a few hours until the pain ceases.

**100 Plasters, ONE DOLLAR.**

**PREPARED ONLY BY THE**

NOVELTY PLASTER WORKS, LOWELL, MASS.

**GEO. E. MITCHELL, Founder and Sole Proprietor.**

**Established in 1864.**

### Second-Hand and Shopworn Dental Engines, Lathes, Etc.

### DENTAL ENGINES.

One S. S. White Engine, old style, good order, $25.00.
One S. S. White Engine, Large Wheel, good order, $30.00.
One Johnston Engine No. 5 H. P., good order, $25.00.
One Johnston Engine No. 6 H. P., $30.00.
One Johnston Engine No. 7 H. P., $30.00.
One Johnston Engine No. 4 H. P., $25.00.
One Johnston Engine No. 5 H. P., $27.50.

### DENTAL CHAIRS.

One O. C. White Head Rest, cost $7.50, sell for $5.00, fine order.

One Archer Closed Arm Chair, raising seat; tips back, N. P. spittoon and carrier, $20.

One Harris Chair, newly upholstered in best Maroon Mohair Plush Med. Base, $80; with new N. P. spittoon.

One Archer Cable Chair; fine order; maroon plush; with spittoon and carrier; cost $115.00, will sell for $65.00.

One Wilkerson Chair; maroon plush, spittoon; etc.; fine order; $125.

One Archer No. 6 Chair; maroon plush; as good as new; attached foot stool, spittoon and carrier; cost $78.50, will sell for $65.00.

### CONTENTS.

COMMUNICATIONS.

Pyorrhoea Alveolaris with Practical Cases.................................... 577

PROCEEDINGS.

Ninth International Medical Congress, Washington, D. C., Sept., 1887. 585

EDITORIAL.

SALMAGUNDI.......................................................................... 629

---

### THE DENTAL REGISTER,

A MONTHLY MAGAZINE DEVOTED TO THE INTERESTS OF THE

**DENTAL PROFESSION.**

*Terms Reduced to* $2.00 *Per Year. Single Copies,* 25 *Cents.*

**EDITED BY PROF. J. TAFT, D.D.S.**

—AND—

**Published by SAM'L A. CROCKER & CO., Ohio Dental and Surgical Depot.**

The volume commences in January and closes in December.

The REGISTER being issued monthly is always fresh, therefore Indispensable to the practitioner who desires to keep posted up with the new inventions and improvements which are daily developed. Neither expense nor effort will be spared to give value and variety to its contents. Articles will be illustrated with well executed engravings whenever they will add to the interest or assist to explanation.

Its extensive circulation and frequency of issue make it one of the BEST DENTAL ADVERTISING MEDIUMS in the United States.

All subscriptions must begin with January or July.

Local or special notices, five lines or less, will be inserted for $3 each insertion; over five lines, 50 cts. per line.

All advertising bills payable in advance, or at furthest, after the issue of the first number containing the advertisement. All transient advertisements to be paid for in advance.

☞CONTRIBUTIONS SOLICITED.

Exclusively Editorial Communications should be addressed to the Editor.

The latest number of the REGISTER may be ordered with or without other goods at any time, and all letters should be addressed to

**SAM'L A. CROCKER & CO., Ohio Dental and Surgical Depot, Cincinnati, O.**

